# The effect of maternal educational status, antenatal care and resumption of menses on postpartum contraceptive use in Ethiopia: systematic review and meta-analysis

**DOI:** 10.1038/s41598-023-39719-w

**Published:** 2023-08-04

**Authors:** Natnael Atnafu Gebeyehu, Kirubel Dagnaw Tegegne, Mesfine Wudu Kassaw

**Affiliations:** 1 Department of Midwifery, College of Medicine and Health Science, Wolaita Sodo University, Sodo, Ethiopia; 2 Department of Comprehensive Nursing, College of Medicine and Health Science, Wollo University, Wollo, Ethiopia; 3 School of Nursing, College of Health Science, Woldia University, Woldia, Ethiopia

**Keywords:** Health care, Medical research

## Abstract

The postpartum period is a crucial starting point for the delivery of family planning services. To date, there are numerous primary studies in Ethiopia on postpartum contraceptive use and related factors. However, the results of key variables are inconsistent, making it difficult to use the results to advance the service dimensions of postpartum contraceptive use in the country. Therefore, this systematic review and meta-analysis was required to summarize this inconsistency and compile the best available evidence on the impact of maternal educational status, antenatal care and menstrual resumption on postpartum contraceptive use in Ethiopia. PubMed, Google Scholar, Scopus, Science Direct, and the repositories of online research institutes were searched. Data were extracted with Microsoft Excel and analyzed with the statistical software STATA (version 14). Data on the study area, design, population, sample size, and observed frequency were extracted using the Joanna Briggs Institute tool. To obtain the pooled effect size, a meta-analysis was performed using a weighted inverse variance random effects model. Cochran's Q X^2^ test, and I^2^ statistics were used to test for heterogeneity, estimate the total quantity, and measure the variability attributed to heterogeneity. A mixed-effects meta-regression analysis was performed to identify possible sources of heterogeneity. To examine publication bias, the Eggers regression test and the Beggs correlation test were used at a p-value threshold of 0.001. Of the 654 articles reviewed, 18 studies met the inclusion criteria and were included in this meta-analysis. Overall, the final analysis includes 11,263 study participants. In Ethiopia, postpartum contraceptive use correlated significantly with maternal educational status (OR = 3.121:95% CI 2.127–4.115), antenatal care follow-up (OR = 3.286; 95% CI 2.353–4.220), and return of the mother's menses (OR = 3.492; 95% CI 1.843–6.615). A uniform meta-regression was performed based on publication year (p = 0.821), sample size (p = 0.989), and city of residence (p = 0.104), which revealed that none of these factors are significant. The use of postpartum contraceptives was found to be better among mothers who are educated, attended antenatal appointments, and resumed their menstrual cycle. Based on our research, we strongly recommended that antenatal care use and maternal educational accessibility need to improve. For family planning professionals, removing barriers to menstruation resumption should be a key priority.

## Introduction

Pregnancy and childbirth-related complications are the leading cause of death and morbidity in women of childbearing age^1^. It is estimated that 295,000 mothers died worldwide in 2017, 94% of which occurred in resource-constrained areas. When the majority of its causes are preventable, Sub-Saharan Africa accounts for about 66% of maternal deaths^2^. Ethiopia had a pregnancy-related maternal mortality rate of 412 per 100,000 live births^3^.

Family planning is widely recognized as a crucial strategy to prevent maternal and infant morbidity and mortality^4^. In countries with high birth rates, promoting family planning can prevent 32% of all maternal deaths and almost 10% of all child deaths^5^. The prevention of unplanned pregnancies and pregnancies in close succession within the first 12 months after birth is referred to as postpartum family planning (PPFP)^6–8^. This involves starting and using contraceptives in the first year after birth^9^. However, due to the low use of family planning in the postpartum period, there are 80 million unwanted pregnancies worldwide^10^. There is a 40% unmet need for family planning among postpartum mothers in sub-Saharan Africa^11^. Since two-thirds of maternal and neonatal mortality occur in the postpartum period^12^, timely family planning within one year of delivery can help prevent these adverse effects^13,14^.

Although 95% of women plan to delay pregnancy by at least two years, postpartum use of modern contraceptives varies significantly between countries, ranging from 73.5% in Zambia to 4% in Pakistan^15–17^. Studies in Ethiopia showed that the postpartum use of modern contraceptives varied between 80.2% in Addis Ababa and 10.2% in Dabat^18,19^.

There is evidence that around half (47%) of all pregnancies in Ethiopia occur within a short delivery interval of less than 24 months^20^. Chronic malnutrition, stunted growth, miscarriage, stillbirth, artificial abortion, prematurity, low birth weight, and neonatal and maternal death are all associated with short gestational intervals^21–26^. The World Health Organization (WHO) therefore suggested that there should be 24 months between each subsequent pregnancy^27^. Short birth intervals^28^ and low use of contraceptives^22^ are said to contribute to an increased number of unwanted pregnancies in Ethiopia. According to research, 21% of the births took place at short intervals of less than 24 months; the remaining 35% took place between 24 and 35 months^28^. The postpartum period is crucial in this situation, particularly for starting contraception and healthy spacing between births^29–31^.

Since 2002, the Ethiopian government has encouraged and expanded the community-based delivery of family planning services to women on their doorstep as part of its health extension program^32^. Despite these initiatives, there is still a significant (86%) unmet need for contraception in women after childbirth^33^. Because it enables women to space birth properly, addressing the contraceptive problem in the postpartum period has a positive impact on maternal, newborn, infant, and child health survival^34^.

The woman's menstruation and resumption of sexual activity, inability to predict fertility resumption, level of education, and utilization of PPFP counseling are some factors influencing postpartum use^19,35,36^.

Recent meta-analysis studies revealed that the national pooled prevalence of post-partum contraceptive use in Ethiopia ranged from 45.44% to 45.7%^37,38^.Although the previous meta-analysis studies were conducted in Ethiopia, for the most part they failed to demonstrate an association between important variables and contraceptive use after childbirth. In addition, the results for key variables are inconsistent, making it difficult to apply the results to the promotion of postpartum contraceptive use in the country's service sector. It was necessary to pool this inconsistency and summarize the best available data on the effects of maternal educational status, prenatal care and menstrual resumption on postpartum contraceptive use in Ethiopia. The results of this study will provide program planners and policymakers with scientific evidence for service improvement. In addition, it helps healthcare professionals by providing scientific insights for practice.

## Methods

### Data synthesis and reporting

We analyzed the data based on a single measurement result (use in postpartum family planning). The results are presented using a forest plot, text, and tables. This systematic review and meta-analysis study was conducted to determine the effects of maternal educational status, antenatal care, and menstrual resumption on the use of postpartum family planning in Ethiopia using the standard PRISMA checklist guideline^39^ (Supplementary File 1). The protocol of this systematic review and meta-analysis has been registered in the International Prospective Register of Systematic Reviews (PROSPERO), registration number CRD42022357519: Available from: https://www.crd.york.ac.uk/prospero/displayrecord.php?ID=CRD42022357519.

### Search strategy

We considered using adapted PICO questions, i.e., the "PEO" (Population, Exposure, Outcome) format was followed, for explicit presentation of our review question and explicit clarification of the inclusion and exclusion criteria. The following keywords and phrases and/or Medical Subject Headings (MeSH), which were combined using the Boolean operators "OR" and "AND," served as the basis for these queries:

### PECO guide

Population.

All postpartum mothers within up to 12 months of delivery.

Exposure.

Maternal educational status, resumption of menses, and antenatal care visit (at least one visit).

Comparison.

No formal education, no antenatal care visit, and no resumption of menses.

Outcome.

Postpartum contraceptive utilization.

We developed the following review question using the above modified PICO format, to identify as many relevant primary studies as possible:

#### Review question

"What is the effect of maternal educational status, antenatal care, and resumption of menses on postpartum contraceptive use during the postpartum period in Ethiopia?".

Thereafter, primary studies on the effect of maternal educational status, prenatal care, and return of menstruation on the use of family planning after childbirth in Ethiopia were searched using Google Scholar, Science Direct, Scopus, EMBASE, and PubMed using the review questions mentioned above. The Bepress legal repository and the Social Science Research Network (SSRN) were our best sources for finding unpublished articles and papers. Therefore, we retrieved gray literature from the Addis Ababa University institutional research repository. The search was conducted using the following keywords and search terms “Contraception”, “Family planning”, “Contraceptive”, “Utilization”, “Use”, “Postpartum mothers”, “Postpartum period”, “Post-delivery”, “Puerperal period”, “Antenatal care”, “Educational status”, “Literacy”, “Returning of menses”, “Resumption of menses”, and “Ethiopia”. The search terms were used individually and in combination with Boolean operators such as “OR” or “AND”. The search was conducted from June 1, 2022, to July 4, 2022.

### Study outcome

The use of family planning services to prevent close and unintended births within the first year after birth is referred to as postpartum contraception^40^.

### Inclusion and exclusion criteria

Only studies from Ethiopia that examined the prevalence of postpartum use of modern contraceptives and its determinants were included in this review. Research and screenings were conducted for both previously published and unpublished articles. All types of observational studies (cross-sectional studies, case–control studies, and cohort studies) have been discussed in this article. In addition, articles published only in English and for which the full text was available were also included. All studies that had been published in the form of journal articles, master's theses, or dissertations by the time the data analysis was completed were included. Therefore, studies that provided data on the association between postpartum family planning and maternal educational status or the association between postpartum contraceptive use and antenatal care, or the association between menstrual resumption and postpartum contraceptive use were included. We excluded works with duplicate reports, studies with qualitative research designs, and studies from other countries. This study used the COCOPOP (Condition, Context, and Population) framework to assess the adequacy of included articles. The study population (POP) consisted of postpartum women; the condition (CO) was the effect of maternal literacy, prenatal care, and menstrual resumption on the use of modern postpartum contraception, and the context (CO) included only studies conducted in Ethiopia.

### Quality assessment

Two authors (NAG and KDT) independently assessed the standard of the studies using the standardized quality assessment checklist of the Joanna Briggs Institute (JBI)^41^. The disagreements raised during the quality assessment were resolved through a discussion led by the third author (MW). Eventually, the dispute was settled and an agreement was reached. The critical analysis checklist contains eight parameters with the options Yes, No, Unclear, and Not Applicable. The parameters address the following questions: (1) Where were the criteria for inclusion in the sample clearly defined? (2) Were the study participants and thus the environment described in detail? (3) Was the exposure measurement result valid and reliable? (4) Were the main objective and standard criteria used to measure the event? (5) Were confounding factors identified? (6) Were strategies given to influence confounding factors? (7) Were the outcomes actually and reliably measured? And (8) Was the statistical analysis appropriate? Studies were considered low-risk if they scored 50% or more on the quality assessment indicators, as indicated in a supplemental file (Supplementary File 2).

### Risk of bias assessment

The bias assessment tool developed by Hoy et al.^42^, which consists of 10 items and measures four bias domains as well as internal and external validity, was used by two authors (NAG and KDT) to independently assess the risk of bias in the included studies. Any disagreements that arose during the risk of bias assessment were resolved through a discussion led by the third author (MW). Eventually, the dispute was settled and an agreement was reached. The first four items (items 1 to 4) assess the presence of selection bias, non-response bias, and external validity. The other six items (items 5–10) assess the presence of a measure of bias, analysis-related bias, and internal validity. Therefore, studies that answered "yes" to eight or more of the ten questions were classified as having a low risk of bias. If studies that received yes to six to seven of the ten questions were classified as moderate risk, studies that received yes to five or fewer of the ten questions were classified as high risk, as reported in a supplemental file (Supplementary File 3).

### Data extraction

Microsoft Excel spreadsheet (2016) and STATA software version 14 were used for data extraction and analysis. Visual inspection and double entry were used to assess the accuracy of the data entered into Excel spreadsheets. The data checker visually examines the entries and compares them with the original paper sheets. In a partner read-aloud, one author reads the paper data sheets aloud while the other author reviews the entries. In double entry, the data checker enters the data a second time; the computer compares the first and second entries to make sure they match and also identifies values that are outside the permitted range. Two authors (NAG and MWM) independently extracted all relevant data using a standardized data extraction format from the Joanna Briggs Institute. The disagreements raised during the data extraction were resolved through a discussion led by the third author (KDT). Eventually, the dispute was settled and an agreement was reached. The data automation tool was used due to the lack of a paper form (manual data) in this study. The first author's name, year of publication, study region, study setting, study design, sample size, the unadjusted odd ratio for factors (educational status, antenatal care, and menstrual resumption), and the quality of each paper was extracted.

### Data analysis

After extraction of all relevant results in a Microsoft Excel spreadsheet, the data were exported to STATA software version 14 for analysis. To obtain a pooled OR, a meta-analysis was performed using a weighted inverse variance random effects model. In addition, a cumulative meta-analysis was performed to illustrate the trend of evidence regarding the impact of maternal educational status, prenatal care, and menstrual resumption on postpartum family planning. To get the publication bias under control, the trim-and-fill approach was adopted by Duval and Tweedies^43^. To test for heterogeneity, estimate the degree of total/residual heterogeneity, and measure variability due to heterogeneity, Cochran's Q X2 test and I2 statistic were used^44^. Study area, sample size, and variations in the year of publication were examined for their effects on heterogeneity between studies using a univariate meta-regression analysis^45^.

### Patient and public involvement

The authors (NA and KD) collaborated with public health experts and researchers from previous studies to design the research questions and outcome metrics. Patients and study participants were not involved in the design or analysis of the study as it was a systematic review and meta-analysis based on published data. The results of this study will be shared with patients and study participants through health education about the factors affecting contraceptive use after childbirth and dissemination of key findings through leaflets in the local language.

## Results

### Search results

**Figure 1:**
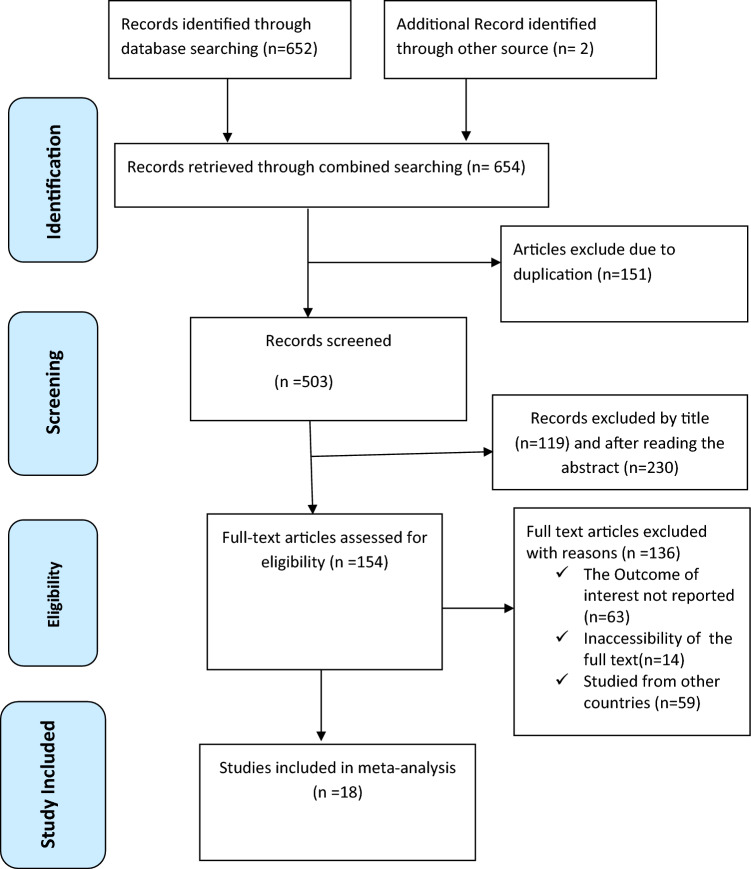
PRISMA flow chart displays the article selection process for the effect of maternal education, antenatal care visit and resumption of menses on postpartum contraceptive use in Ethiopia.

In total, we obtained 654 articles from international online databases (PubMed, Scopus, EMBASE, Science Direct, and Google Scholar) and gray literature from the Addis Ababa University repository. After removing duplicate studies, we received 503 studies that were selected for full title and abstract screening. Of these, 340 studies were excluded based on title and abstract, and the remaining 154 articles were assessed as full-text articles. Subsequently, 136 articles were excluded for other reasons after checking the full text. Finally, 18^35,36,46–61^ studies met the inclusion criteria and were used in this meta-analysis: 8 studies^48,50,53–58^ examined the association between ANC attendance and PPFP use, 9 studies^35,36,46–52^ reported the association between maternal educational status and PPFP use, while 12 studies^35,36,46,50–53,57–61^ reported the association between menstrual resumption and PPFP use. The PRISMA diagram flowchart for the item selection process is shown in Fig. 1. In this meta-analysis, a study could report more than one outcome measure or related factors.

### Study characteristics

As shown in Table 1, 18 studies reported the association between ANC attendance, maternal educational status, menstrual resumption, and postpartum family planning in 11,263 mothers. Of these studies, five were conducted in the Amhara region^36,49,51,58,60^; five in Southern Nations Nationalities and Peoples (SNNPR) regions^36,48,51,58,60^, three in the Oromia region^52,54,56^, two in Tigray region^35,55^, two in Addis Ababa^53,61^ and a study is being carried out at national level^48^. All included studies were conducted using a cross-sectional study design. Fourteen studies were community-based studies, while the remaining four studies were health facility studies. Fifteen studies were conducted in mothers within the previous 12 months, while three studies were conducted in mothers six weeks after delivery. The sample size ranged from 248 to 2304. All studies were assessed using the Joanna Briggs Institute (JBI) Quality Assessment Checklist and found to be low risk.

**Table 1:** A descriptive summary of 18 studies included in the meta-analysis for estimation of the effect of maternal educational status, antenatal care visit, and resumption of menses on postpartum contraceptive use in Ethiopia, 2020.

Author/year	Study area	Study Region	Study setting	Study design	Sample size	Quality	Association of educational status with PPFP	Association of antenatal care with PPFP	Association of resumption of menses with PPFP	Study participant’s characteristic’s
Wassihun etal. /2021^36^	Arbaminch town	SNNPR	Health facility	Cross-sectional	408	Low-risk	1.66 (1.28–3.55)	Not reported	Not reported	After 6 weeks of delivery
Dagnaw et al. /2020^48^	National	National	Community	Cross-sectional	2304	Low-risk	1.79 (1.04–3.10)	2.59 (1.43–4.69)	Not reported	Within 12 months of delivery
Belete AG/un-pub^49^	Injibara town	Amhara	Community	Cross-sectional	400	Low-risk	5.02 (1.53–16.47)	Not reported	Not reported	Within 12 months of delivery
Zeleke Girma Abate et al. /2021^50^	Dila town	SNNPR	Community	Cross-sectional	293	Low-risk	5.03 (3.24–6.97)	5.81 (1.89–7.58)	6.03 (2.92–10.23)	After 6 weeks of delivery
Gejo et al. /2019^37^	Hosana Town	SNNPR	Health facility	Cross-sectional	368	Low-risk	0.26 (0.09–0.744)	Not reported	8.48 (3.072–23.228)	Within 12 months of delivery
Wassachew Ashebir /2020^47^	Burie district	Amhara	Community	Cross-sectional	681	Low-risk	0.15 (0.03–0.71)	Not reported	0.39 (0.25–0.50)	Within 12 months of delivery
Abraha TH et al. /2017^35^	Axum town	Tigray	Community	Cross-sectional	590	Low-risk	4.25 (1.29–14.00)	Not reported	6.35 (3.14–13.39)	Within 12 months of delivery
Nibret Mihret et al. /2020^51^	Addis Ababa	Amhara	Community	Cross-sectional	402	Low-risk	2.99 (1.72–5.19)	Not reported	4.76 (3.03–7.48)	Within 12 months of delivery
Solomon Girma Nigusie et al. /2019^52^	Delomena woreda	Oromia	Community	Cross-sectional	505	Low-risk	5.5 (2.13–14.22)	Not reported	6.12 (2.58–14.52)	Within 12 months of delivery
Tafa L/2021^53^	Addis Ababa	Addis Ababa	Health facility	Cross-sectional	625	Low-risk	Not reported	4.96 (1.58–15.64)	1.75 (1.11–2.76)	Within 12 months of delivery
Teka et al. /2018^54^	Wollega	Oromia	Community	Cross-sectional	603	Low-risk	Not reported	2.93 (1.08–7.94)	Not reported	Within 12 months of delivery
Abreha et al. /2018^55^	Not reported	Tigray	Community	Cross-sectional	1109	Low-risk	Not reported	2.1 (1.90–4.20)	Not reported	Within 12 months of delivery
Bushura Negaso /un-pub^56^	Ambo	Oromia	Community	Cross-sectional	385	Low-risk	Not reported	9.71 (3.83–24.61)	Not reported	Within 12 months of delivery
Mahlet Getachew/un-pub^57^	Butajira district	SNNPR	Community	Cross-sectional	420	Low-risk	Not reported	3.81 (1.53–9.51)	3.71 (1.93–7.12)	Within 12 months of delivery
Abera et al. /2015^58^	Gondar town	Amhara	Community	Cross-sectional	703	Low-risk	Not reported	5.76 (2.18–15.2)	8.32 (5.27–13.14)	Within 12 months of delivery
Mesfin Yesigat et.al/2022^59^	Arbaminch town	SNNPR	Community	Cross-sectional	416	Low-risk	Not reported	Not reported	5.3 (3.12–9.15)	Within 12 months of delivery
Demie TG/2018^60^	Debre-Berhan town	Amhara	Health facility	Cross-sectional	248	Low-risk	Not reported	Not reported	1.907 (5.01–20.174)	Mothers after 6 weeks of delivery
Gebremedhin et al./2018^61^	Addis Ababa	Addis Ababa	Community	Cross-sectional	803	Low-risk	Not reported	Not reported	2.12 (1.37–3.41)	Within 12 months of delivery

### Meta-analysis

#### Postpartum family planning utilization in Ethiopia

**Figure 2:**
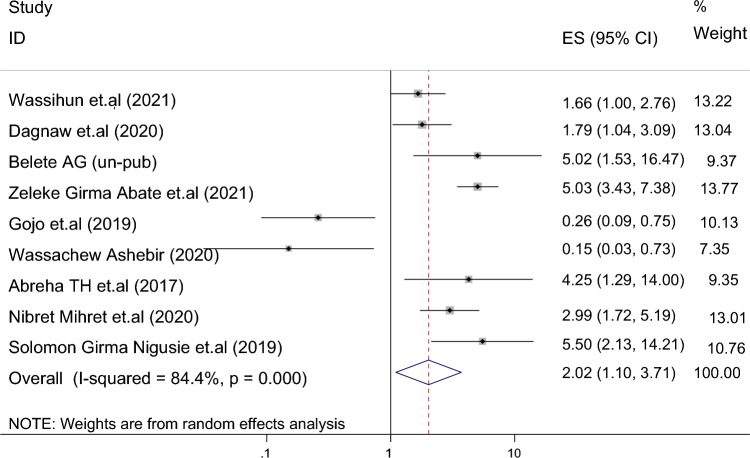
Forest plot showing the results from a cumulative meta-analysis of studies examining the effect of maternal education on postpartum contraceptive use.

**Figure 3:**
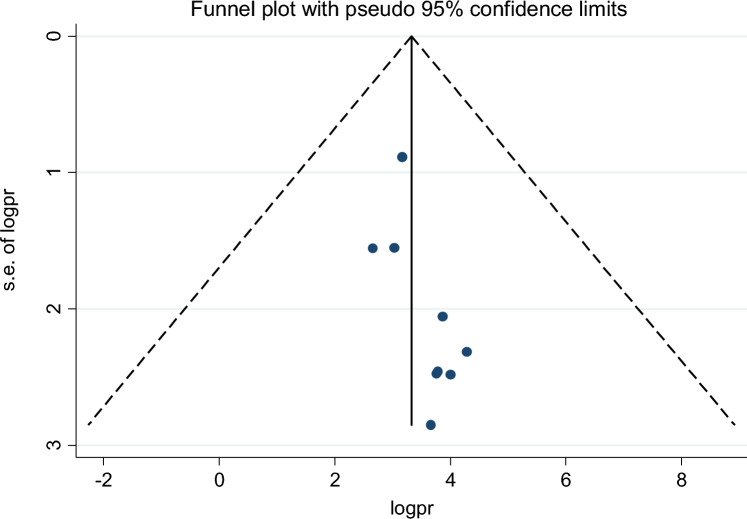
Funnel plot showing the distribution of studies examining the effect of maternal education on postpartum contraceptive use.

**Figure 4:**
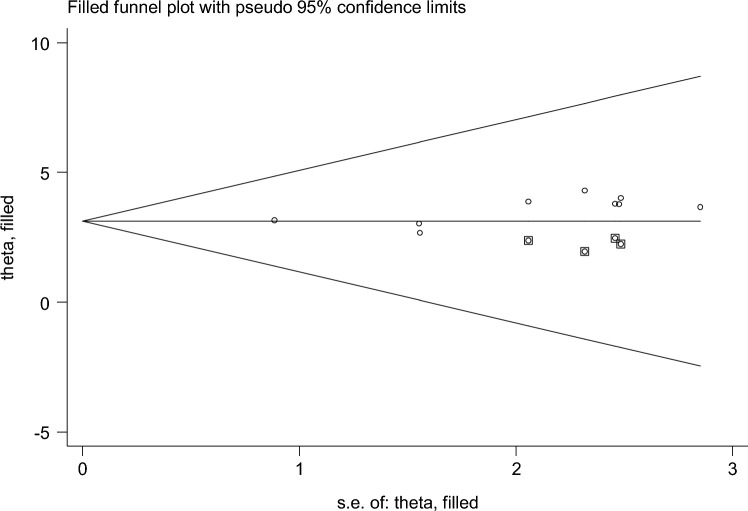
Trim and fill method of analysis for studies examining the effect of maternal educational status on postpartum contraceptive use.

Of the 18 studies included, 9 studies reported the association between maternal educational status and the use of family planning after childbirth in 5951 mothers (Table 1). The pooled odds ratio for maternal educational status was 2.108 (95% CI 1.097–3.710), I^2^ = 84.4%, p = 0.000 (Fig. 2). There is a funnel plot asymmetry in visual observation (Fig. 3). We also ran Eggers' regression test and Beggs' correlation test, which gave p = 0.049 and p = 0.037, respectively. Since significant publication was found, we performed a Duval and Tweedie trim-and-fill analysis and calculated a new effect size for maternal educational status (OR = 3.121, 95% CI 2.127–4.115), I^2^ = 90 0.2% after inclusion of imputed studies (i.e. estimated number of missing studies = 4) (Fig. 4). Therefore, mothers with an elementary or higher education were three times more likely to engage in postpartum family planning than mothers with no formal education.

**Figure 5:**
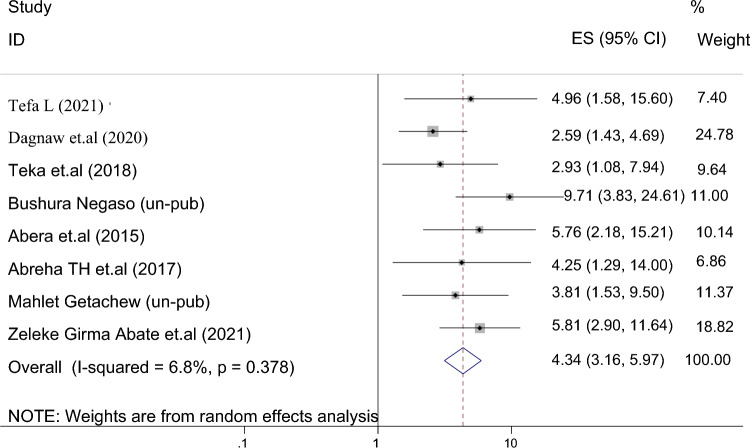
Forest plot displaying studies examining the effect of antenatal care on postpartum contraceptive use in Ethiopia.

**Figure 6:**
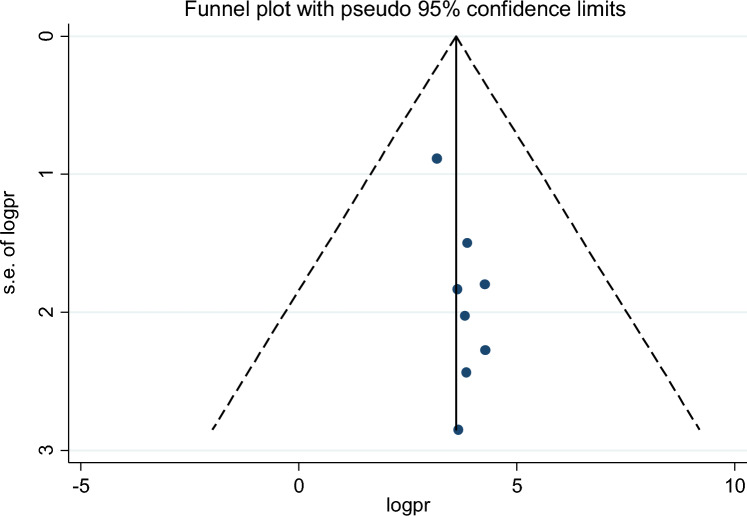
Funnel plot displaying the distribution of studies examining the effect of antenatal care on postpartum contraceptive use in Ethiopia.

**Figure 7:**
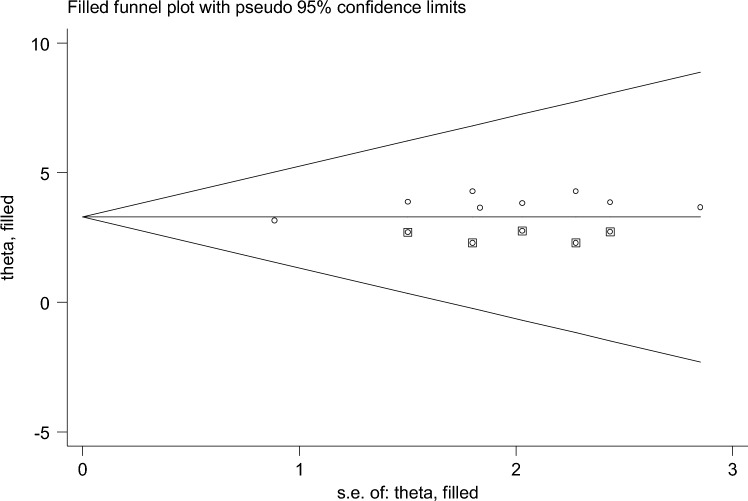
Trim and fill method of analysis for studies examining the effect of antenatal care visit status on postpartum contraceptive use.

Eight studies reported the association between antenatal visits and the use of family planning after childbirth in 6442 mothers. The pooled odds ratio for antenatal visits was 4.340 (95% CI 3.157–5.966), I^2^ = 60.8%, P = 0.378 (Fig. 5). The funnel plot shows an asymmetric distribution of the studies (Fig. 6). Because Egger's test (p = 0.024) and Begg's test (p = 0.032) showed significant publication bias, we performed Duval and Tweedie trim-and-fill analysis and calculated a new effect size for visits to the antenatal care (OR = 3.286, 95%). CI 2.353–4.220), I^2^ = 73.2% after inclusion of imputed articles (i.e. estimated number of missing studies = 5) (Fig. 7). Thus, mothers who had at least one and above antenatal care visit were 3 times more likely to utilize postpartum family planning as compared to mothers who had no antenatal care visit.

**Figure 8:**
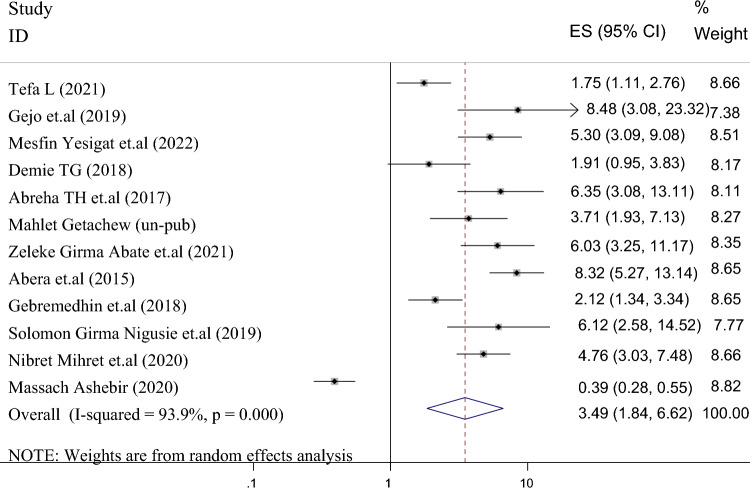
Forest plot showing the results from a cumulative meta-analysis of studies examining the effect of resumption of on postpartum contraceptive use.

**Figure 9:**
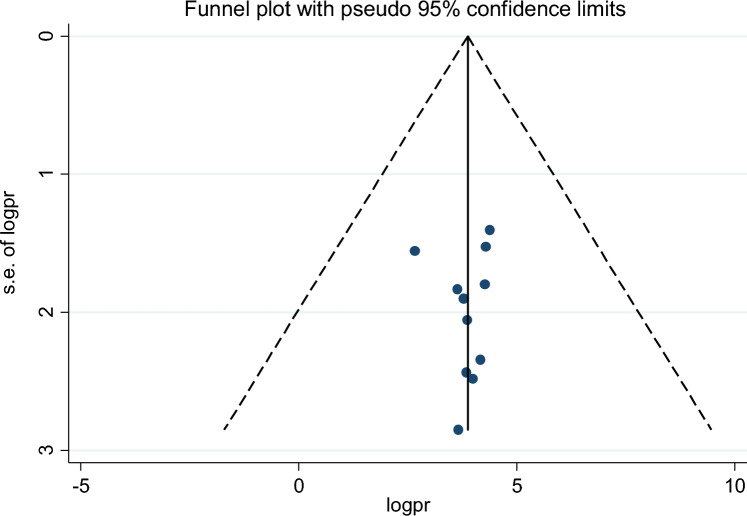
Funnel plot displaying the distribution of studies examining the effect of resumption of menses on postpartum contraceptive use in Ethiopia.

Twelve studies in 6059 mothers showed an association between the return of menstruation and the use of family planning after childbirth. The pooled odds ratio for menstrual resumption was 3.492 (95% CI 1.843–6.615), I2 = 93.9%, P = 0.000 (Fig. 8). The funnel chart shows a symmetrical distribution of the studies (Fig. 9). The Egger test (p = 0.312) and the Begg test (p = 0.533) also showed no evidence of publication bias. As a result, the odds of postpartum family planning use were 3.5 times higher among women who had resumption of menses than their counterparts. We also conducted a Univar ate meta-regression by considering the year of publication, sample size, and residence in Ethiopia as covariates to identify the possible sources of heterogeneity, and unfortunately, none was found to be statistically significant (Table 2).

**Table 2:** Meta-regression analysis of factors affecting between-study heterogeneity.

Heterogeneity source	Coefficient’s	Standard error	p-value
Publication year	127.6568	553.1591	0.821
Sample size	3.894071	275.9929	0.989
Residence	4.823553	1.403627	0.104

## Discussion

Mothers can begin family planning methods in the postpartum period, although this is typically a crucial window to seek family planning services^28,62^. One of the proposed public health interventions to reduce maternal and child morbidity and mortality rates is postpartum family planning services^63,64^. The Ethiopian Health Sector Transformation Plan (EHSTP)^65^ and the Sustainable Development Goals (SDGs)^66^ on maternal and child health can both be achieved by identifying the factors affecting the availability of postpartum family planning strategies during the postnatal period affect phase. Therefore, the purpose of this systematic review and meta-analysis was to assess the impact of maternal educational status, prenatal care follow-up, and menstrual resumption on postpartum family planning engagement in Ethiopia. The main findings were that the use of family planning after childbirth was significantly associated with maternal educational status, follow-up for prenatal care, and women's resumption of menses after childbirth.

In contrast to mothers who experienced amenorrhea after childbirth, our data showed that mothers who resumed their menstrual cycle were significantly more likely to engage in family planning after childbirth. This is consistent with studies conducted in Malawi, Nepal, India, Malawi, and Nigeria^67–69^ and with Demographic Health Survey analyses from 27 different countries^70^. Studies with meta-analyses conducted in Ethiopia^38^ and in low- and middle-income countries^70^ also supported this judgment. This could be because women feel more vulnerable to unwanted pregnancies after their periods return. As a result, they are more likely to start using postpartum contraceptives. Knowing that a woman will ovulate before her first period after childbirth may also contribute to pregnancy^32,71^ and provides further support for this conclusion.

We also found that mothers who had at least one prenatal visit were three times more likely to use contraception after childbirth than mothers who did not. This conclusion is consistent with research from Ethiopia^38^, results from 17 countries for USAID^70^, Mexico^72^, India^73^, and Bangladesh^74^, results from 17 countries for USAID^70^, and an Ethiopian meta-analysis study^38^. This may be because women who received ANC were more likely to have access to information about birth spacing and the risks of a short birth for both mother and child. In addition, antenatal visits provide an opportunity to engage clients and providers in counseling sessions, which helps improve health-focused behaviors and contraceptive use.

Finally, we have shown that maternal educational status is significantly correlated with postpartum family planning use, consistent with other research from India^75^, Uganda^76^, Ghana^74^, and meta-analysis studies from Ethiopia^37^ countries with low and middle income^76^. As mothers become more educated, they may be more likely to seek medical care, understand the pros and cons of contraception, and have access to accurate information about fertility and contraception. Therefore, strengthening maternal education helps empower women to make informed decisions about their fertility and promotes the health of mothers and their children.

The PRISMA guidelines for literature searches were used to conduct this meta-analysis. In addition, the Egger statistical regression test and the Begg correlation test were used to calculate publication bias. We used the Joanna Briggs Institute Quality Assessment Checklist. The data may be useful for aspiring researchers, family planning specialists, and healthcare decision-makers as it is the first study of its kind in Ethiopia. Another benefit of this meta-analysis is that it includes both published and unpublished studies. In addition, this study has shortcomings. Because all included studies were observational, there is less solid evidence of a causal relationship. The risk of overlooking relevant research cannot be eliminated, even though we used extensive search tactics and the result may not be nationally representative.

Several analyses show significant differences between studies when using the traditional heterogeneity test approach. A meta-regression analysis was used to carefully analyze the course of heterogeneity. Because the research region, sample size, and year of publication varied, caution should be exercised when interpreting the results. In addition, the dose–response relationship between the frequency of ANC visits and use for postpartum family planning has not been investigated. Research has reported the association between maternal educational status, ANC attendance, and use of family planning after childbirth, however, significant publication bias was also found in these studies. To correct for publication bias and provide an unbiased estimate, we performed a Duval and Tweedie trim-and-fill analysis; however, the result should be interpreted with caution.

## Conclusion

In conclusion, we found that mothers who are educated, have attended antenatal care, and have resumed their menstrual cycle are more likely to use postpartum contraception. We strongly advocated for increased access to maternal education and antenatal care follow-up service. Additionally, overcoming menstrual cycle problems is the top priority for family planning providers.

### Implications of the findings

The impact of the study on various scientific communities and the general public is as follows it is based on secondary data. Ethiopia and other developing countries have low rates of postpartum contraceptive use. This study suggests that maternal educational level, prenatal care, and the return of menstruation have an impact on postpartum contraceptive use. The results of this study, therefore, encourage researchers to conduct further, more detailed investigations to determine the association between postpartum contraceptive use and other characteristics not considered in this analysis. The third conclusion of this study is that as the use of postpartum family planning services increases, the leading causes of maternal deaths—unintended pregnancies, short-term pregnancies, unsafe abortions, and other maternal morbidity—will decrease.

### Supplementary Information

Supplementary Table 1.

Supplementary Table 2.

Supplementary Table 3.

## Data Availability

All relevant data are within the Manuscript and its Supporting Information files.

## Abbreviations

OR: Odd ratio
CI: Confidence interval
PPFP: Postpartum family planning
