# Supplementary material for: The effect of maternal educational status, antenatal care and resumption of menses on postpartum contraceptive use in Ethiopia: systematic review and meta-analysis
Source: Sci Rep. 2023 Aug 4;13:12655. doi: 10.1038/s41598-023-39719-w (PMC10403499; doi:10.1038/s41598-023-39719-w)
Supplement: Supplementary file 2 — Supplementary Table 2. [file 41598_2023_39719_MOESM2_ESM.docx]

Table2. Quality assessment for the included Studies

| Item | Clearly defined inclusion | Describe study setting and participant | Valid and reliable exposure measurement | Objective and standard criteria for measurement | Identified confounder | Strategies to deal with confounders | Valid and reliable outcome measurement | Appropriate statically analysis | No of ‘yes’s ‘ |
| --- | --- | --- | --- | --- | --- | --- | --- | --- | --- |
| Wassihun et.al | Yes | Yes | No | Yes | Yes | No | Yes | Yes | 6/8=75 |
| Dagnaw et.al | Yes | Yes | Yes | Yes | No | No | Yes | Yes | 6/8=75 |
| Belete AG | Yes | Yes | No | Yes | Yes | No | Yes | Yes | 6/8=75 |
| Zeleke Girma Abate et.al | Yes | Yes | No | Yes | Yes | Yes | Yes | Yes | 7/8=87.5 |
| Gejo et.al | Yes | Yes | No | Yes | Yes | Yes | Yes | Yes | 7/8=87.5 |
| Wassachew Ashebir | Yes | Yes | Yes | Yes | Yes | No | Yes | Yes | 7/8=87.5 |
| Abraha TH et.al | Yes | Yes | No | Yes | Yes | Yes | Yes | Yes | 7/8=87.5 |
| Nibret Mihretie et.al | Yes | Yes | Yes | Yes | No | No | Yes | Yes | 6/8=75 |
| Solomon Girma Nigusie et.al | Yes | Yes | No | Yes | Yes | No | Yes | Yes | 6/8=75 |
| Tefa L | Yes | Yes | Yes | Yes | Yes | No | Yes | Yes | 7/8=87.5 |
| Teka et.al | Yes | Yes | Yes | Yes | No | No | Yes | Yes | 6/8=75 |
| Abreha et.al | Yes | Yes | No | Yes | Yes | Yes | Yes | Yes | 7/8=87.5 |
| Bushura Negasa | Yes | Yes | Yes | Yes | No | No | Yes | Yes | 6/8=75 |
| Mahlet Getachew | Yes | Yes | Yes | Yes | Yes | No | Yes | Yes | 7/8=87.5 |
| Abera et.al | Yes | Yes | Yes | Yes | No | No | Yes | Yes | 6/8=75 |
| Mesfine Yesigat et.al | Yes | Yes | Yes | Yes | Yes | No | Yes | Yes | 7/8=87.5 |
| Demie TG | Yes | Yes | Yes | Yes | No | No | Yes | Yes | 6/8=75 |
| Gebremedhin et.al | Yes | Yes | Yes | Yes | Yes | No | Yes | Yes | 7/8=87.5 |

Methodological quality assessment of included studies using Joanna Brigg's Institute quality appraisal criteria scale (JBI). The eight-item questions assessing inclusion criteria, study setting and participant, exposure measurement, objectives, confounder, statically analysis, outcome measurement, and dealing confounder were used.
