# Supplementary material for: The effect of maternal educational status, antenatal care and resumption of menses on postpartum contraceptive use in Ethiopia: systematic review and meta-analysis
Source: Sci Rep. 2023 Aug 4;13:12655. doi: 10.1038/s41598-023-39719-w (PMC10403499; doi:10.1038/s41598-023-39719-w)
Supplement: Supplementary file 3 — Supplementary Table 3. [file 41598_2023_39719_MOESM3_ESM.docx]

Table 3. Assessment of risk of bias for the included studies

| Item | External validity | | | | Internal validity | | | | | |  | |
| --- | --- | --- | --- | --- | --- | --- | --- | --- | --- | --- | --- | --- |
|  | Representativeness s of the target population | Representativeness s of the sampling frame | Radom sampling g or census | Minimal response e bias | Data were collected d directly | Acceptable e case definition used in the study | Valid and reliable measurement t | The same mode of data collection n for all study subject | Appropriate e length of prevalence period for parameter of interest | Appropriate numerators and denominator s of interest | No of yes | Summary of risk of bias |
| Wassihun et.al | Yes | Yes | No | Yes | Yes | No | Yes | Yes | Yes | Yes | 8 | Low-  risk |
| Dagnaw et.al | Yes | Yes | Yes | Yes | No | No | Yes | Yes | Yes | Yes | 8 | Low risk |
| Belete AG | Yes | Yes | No | Yes | Yes | No | Yes | Yes | Yes | Yes | 8 | Low- risk |
| Zeleke Girma Abate et.al | Yes | Yes | No | Yes | Yes | Yes | Yes | Yes | Yes | Yes | 9 | Low risk |
| Gejo et.al | Yes | Yes | No | Yes | Yes | Yes | Yes | Yes | Yes | Yes | 9 | Low- risk |
| Wassachew Ashebir | Yes | Yes | Yes | Yes | Yes | No | Yes | Yes | Yes | Yes | 9 | Low- risk |
| Abraha TH et.al | Yes | Yes | No | Yes | Yes | Yes | Yes | Yes | Yes | Yes | 9 | Low- risk |
| Nibret Mihretie et.al | Yes | Yes | Yes | Yes | No | No | Yes | Yes | Yes | Yes | 8 | Low- risk |
| Solomon Girma Nigusie et.al | Yes | Yes | No | Yes | Yes | Yes | Yes | No | Yes | Yes | 8 | Low- risk |
| Tefa L | Yes | Yes | Yes | Yes | Yes | No | Yes | Yes | Yes | Yes | 9 | Low- risk |
| Teka et.al | Yes | Yes | Yes | Yes | No | No | Yes | Yes | Yes | Yes | 8 | Low- risk |
| Abreha et.al | Yes | Yes | No | Yes | Yes | Yes | Yes | Yes | Yes | Yes | 9 | Low – risk |
| Bushura Negasa | Yes | Yes | No | Yes | Yes | No | Yes | Yes | Yes | Yes | 8 | Low-  risk |
| Mahlet Getachew | Yes | Yes | No | Yes | Yes | No | Yes | Yes | Yes | Yes | 8 | Low-  risk |
| Abera et.al | Yes | Yes | No | Yes | Yes | Yes | Yes | Yes | Yes | Yes | 9 | Low- risk |
| Mesfine Yesigat et.al | Yes | Yes | Yes | Yes | No | No | Yes | Yes | Yes | Yes | 8 | Low- risk |
| Demie TG | Yes | Yes | No | Yes | Yes | Yes | Yes | Yes | Yes | Yes | 9 | Low- risk |
| Gebremedhin et.al | Yes | Yes | Yes | Yes | No | No | Yes | Yes | Yes | Yes | 8 | Low risk |

Risk of bias assessment for the included studies. The ten-item questions of which four items assess external and six items assess internal validity were used
